# Perception of respectful maternity care and birth satisfaction among postpartum women: a cross-sectional study in a baby-friendly hospital**

**DOI:** 10.1590/1806-9282.20260558

**Published:** 2026-08-10

**Authors:** Ayca Erduran, Nuray Egelioğlu Cetişli

**Affiliations:** 1 Dokuz Eylül University – İzmir, Türkiye.; 2 Izmir Katip Celebi University, Faculty of Health Science, Department of Obstetrics and Gynecology Nursing – İzmir, Türkiye.

**Keywords:** Labor, Maternal health services, Nursing. Obstetrics.

## Abstract

**OBJECTIVE:**

The aim of this study was to examine the relationship between perceptions of respectful childbirth care and birth satisfaction among women giving birth in a baby-friendly hospital and to identify factors associated with these outcomes.

**METHODS:**

A cross-sectional design was used to examine women who gave birth in the obstetrics and gynecology clinic of a baby-friendly university hospital in Türkiye. In total, 200 postpartum women meeting the inclusion criteria were included in the study. Data were collected using the Personal Information Form, Perception of Respectful Maternity Care Scale, and Birth Satisfaction Scale. Data were analyzed using descriptive statistics, nonparametric tests, Pearson correlation, and multiple regression analysis.

**RESULTS:**

Multiple regression models explained 8.1–19.4% of the variance in the perception of respectful childbirth care and 6.8–13.3% of the variance in birth satisfaction. A moderate and statistically significant correlation was found between the perception of respectful maternal care and childbirth satisfaction (r=0.464, p<0.001).

**CONCLUSION:**

A higher perception of respectful maternity care is associated with greater birth satisfaction. It is recommended that nurses and other healthcare professionals adopt this care model, receive related training, and practice woman-centered care.

## INTRODUCTION

Respectful maternity care (RMC) may enhance birth satisfaction by fostering trust, reducing fear and stress, and strengthening women’s sense of empowerment and participation during childbirth. For this reason, exploring women’s perceptions of RMC and examining how these perceptions are associated with birth satisfaction is essential for evaluating maternity care quality beyond clinical indicators alone^1,2^. Studies in the literature on this subject have reported that women experienced physical and verbal violence and discrimination during childbirth, that their consent was not obtained for medical procedures, that they experienced communication deficiencies, that arrangements should be made to ensure their privacy, and that the vast majority evaluated their childbirth experiences negatively^3,4,5,6^.

Baby-friendly hospitals (BFH), supported by the WHO and UNICEF, aim to promote breastfeeding, mother–infant bonding, and woman-centered care practices. Beyond breastfeeding outcomes, BFH standards emphasize informed decision-making during childbirth, respectful communication, privacy, and emotional support^7^. These principles closely align with the core components of RMC^1^. However, although BFH status provides an institutional framework that may facilitate RMC, there is limited evidence regarding how women perceive such care and how these perceptions influence birth satisfaction^8,9^. Understanding how women perceive childbirth care and how these perceptions relate to birth satisfaction is crucial for evaluating the effectiveness of policy-driven care models in improving women’s childbirth experiences. Moreover, this understanding is essential for enhancing the quality of intrapartum services and guiding patient-centered care interventions^6^. Identifying sociodemographic and antenatal factors associated with perceptions of RMC and birth satisfaction can provide valuable insights for healthcare professionals and policymakers aiming to improve the quality of maternal care. To date, no study in Turkey has simultaneously examined perceptions of RMC and birth ­satisfaction within a BFH setting using multivariable regression models. Therefore, the aim of this study was to examine the relationship between women’s perceptions of RMC and birth satisfaction and to identify sociodemographic and obstetric factors associated with these outcomes in the context of a BFH. Accordingly, the following research questions were addressed:

- What are the perceptions of RMC among women who have given birth?
- What are the levels of birth satisfaction among women who have given birth?
- Which factors influence women’s perceptions of RMC and birth satisfaction?
- Is there a correlation between women’s perceptions of RMC and their birth satisfaction?

## METHODS

This study employed an analytical cross-sectional design. The research followed the STROBE guidelines to ensure methodological rigor and transparency. Ethical approval for the study was obtained from the relevant Ethics Committee (Date: 11.09.2023; IRB:0473).

The study was conducted between December 2023 and December 2024 in the obstetrics and gynecology clinic of a university hospital in İzmir, Turkey, which holds the BFH designation. The study population consisted of all women who gave birth at this hospital during the specified period. The sample size was calculated using the G*Power 3.1.9 statistical software. Based on multiple linear regression analysis, with a medium effect size (0.15), 90% power, and a significance level of 0.05, the required sample size was determined to be 172 postpartum women. To account for potential data loss, the study was completed with 200 mothers who met the inclusion criteria. Mothers who were aged 18 years or older, married, able to read, write, and speak Turkish; had a term singleton birth; had completed the first 24 h postpartum; had no previous diagnosis of any psychiatric disorder; and voluntarily agreed to participate in the study were accepted into the study.

Data were collected by the researcher through face-to-face interviews conducted in the hospital room with mothers who had completed the first 24 h postpartum and met the inclusion criteria. Each interview lasted approximately 20–30 min. To minimize response bias, participants were informed that their responses would remain confidential and would not affect the care they received.

The Personal Information Form included sociodemographic characteristics and obstetric characteristics of the mothers.

The Perception of Respectful Maternity Care Scale (PRMCS) was originally developed by Ayoubi et al. in 2020, and its Turkish validity and reliability study was conducted in 2022. The scale consists of 19 items and three subscales: providing comfort, participatory care, and mistreatment. Total scores range from 19 to 95, with higher scores indicating a more positive perception of RMC. The Cronbach’s alpha coefficient was reported as 0.91 in the original study^10^ and 0.96 in the Turkish validation study^11^; in the present study, the Cronbach’s alpha coefficient was 0.82.

The Birth Satisfaction Scale (BSS) was developed in 2009, revised in 2014, and adapted into Turkish in 2015. This five-point Likert-type scale consists of 30 items. Total scores range from 30 to 150, with higher scores indicating greater birth satisfaction. The Cronbach’s alpha coefficient was reported as 0.62 in the Turkish adaptation study^12^ and was found to be 0.83 in the present study.

Data were analyzed using SPSS version 25.0. The normality of data distribution was assessed using the Kolmogorov-Smirnov test. Multiple linear regression analysis was conducted to identify sociodemographic and obstetric factors associated with perceptions of RMC and birth satisfaction. Regression models were constructed using variables that were found to be significant in univariate analyses and supported by the literature. Regression assumptions—including normality of residuals, homoscedasticity, absence of multicollinearity (variance inflation factor <5), and independence of errors—were assessed and met. The fixed effects of sociodemographic and obstetric factors on total PRMCS and BSS scores were expressed using regression coefficients, adjusted regression coefficients, 95%CIs, and p-values. Relationships between continuous variables were examined using Pearson correlation analysis. All statistical analyses were evaluated at a 95%CI.

## RESULTS

In the study, the mean age of the participants was 32.5±4.3 years, 46% of the women were high school graduates, 55.5% were employed, and 96% had nuclear families. In total, 87% of the women were planned, 77% had received information about the pregnancy and birth process, 91% had not attended childbirth preparation classes, 73% had given birth by cesarean section, and 66% had given birth to a baby girl (Table 1).

**Table 1:** Sociodemographic and obstetric characteristics of the participants (n=200).

Variables	Mean±SD (min–max)
Mean age (years)	32.5±4.3 (22–46)
Educational status	n (%)
Primary school	6 (3.0)
Secondary school	15 (7.5)
High school	92 (46.0)
University	87 (43.5)
Employment status	
Employed	111 (55.5)
Unemployed	89 (44.5)
Family type	
Nuclear family	192 (96.0)
Extended family	8 (4.0)
Planned pregnancy status	
Yes	174 (87.0)
No	26 (13.0)
Mode of birth	
Vaginal	54 (27.0)
Cesarean section	146 (73.0)
Receiving information about pregnancy and birth process	
Yes	154 (77.0)
No	46 (23.0)
Attendance at childbirth preparation classes	
Yes	18 (9.0)
No	182 (91.0)
Infant sex	
Male	68 (34.0)
Female	132 (66.0)

SD: standard deviation.

The mean total score of the participants in the study on the PRMCS was 71.56±7.14, the mean score for the Providing Comfort subscale was 26.42±2.85, the mean score for the Participatory Care subscale was 21.31±4.26, and the mean score for the Mistreatment subscale was 23.84±1.25. Multiple linear regression analysis including sociodemographic variables showed that the model was statistically significant (p=0.001) and explained 8.1% of the variance in perceptions of RMC (R^2^=0.081). Among the associated factors, family type was significantly associated with PRMCS scores (β=-0.204, p=0.005), whereas educational level and employment status were not significantly associated (p>0.05) (Table 2). Multiple linear regression analysis including planned pregnancy status, mode of birth, and infant sex indicated that the model was statistically significant (p<0.001) and explained 19.4% of the variance in perceptions of RMC (R^2^=0.194). Planned pregnancy (β=-0.171, p=0.009) and mode of birth (β=-0.378, p<0.001) were significantly associated with PRMCS scores, whereas infant sex was not (p>0.05) (Table 3).

**Table 2:** Multivariable linear regression analysis of sociodemographic factors associated with perception of respectful maternity care and birth satisfaction.

Scales	Variables	B	SE	β^*^	t	p-value	95%CI for B
Perception of respectful maternity care scale	Constant	74.536	5.381		13.851	<0.001	
	Education (ref: primary school)	1.253	0.772	0.129	1.623	0.106	-0.270 to 2.776
	Employment (ref: unemployed)	-0.454	1.122	-0.032	-0.405	0.686	-2.667 to 1.758
	Family type (ref: nuclear)	-7.413	2.617	-0.204	-2.832	0.005	-12.575 to -2.252
	Model fit	R=0.285, R^2^=0.081; Adjusted R^2^=0.067; F(3.196)=5.796; p=0.001					
Birth satisfaction scale	Constant	87.500	7.636		11.459	<0.001	
	Education (ref: primary school)	2.609	1.152	0.163	2.266	0.025	0.338–4.880
	Family type (ref: nuclear)	-8.220	4.301	-0.162	-2.256	0.025	-18.256 to -1.244
	Model fit	R=0.261, R^2^=0.068; Adjusted R^2^=0.059; F(2.197)=7.215; p=0.001					

B: unstandardized regression coefficient; **β**: standardized regression coefficient; CI: confidence interval. SE: standard error.

*Negative **β** values indicate lower scores compared to the reference category.

The mean total score of the BSS for women who had given birth and were included in the study was 88.59±11.80. Multiple linear regression analysis including sociodemographic variables indicated that the model was statistically significant (p=0.001) and explained 6.8% of the variance in birth satisfaction scores (R^2^=0.068). Educational level (β=0.163, p=0.025) and family type (β=-0.162, p=0.025) were significantly associated with BSS scores (Table 2). Multiple linear regression analysis including birth-related characteristics showed that the model was statistically significant (p<0.001) and explained 13.3% of the variance in birth satisfaction scores (R^2^=0.133). Receiving information about pregnancy and childbirth (β=0.323, p<0.001) and participation in childbirth preparation classes (β=-0.155, p=0.022) were significantly associated with BSS scores (Table 3).

**Table 3:** Multivariable linear regression analysis of obstetric factors associated with perception of respectful maternity care and birth satisfaction.

Scales	Variables	B	SE	β^*^	t	p-value	95%CI for B
Perception of respectful maternity care scale	Constant	88.152	2.700		32.779	<0.001	
	Planned pregnancy status (ref: no)	-3.617	1.361	-0.171	-2.658	0.009	-6.301 to -0.933
	Mode of birth (ref: vaginal)	-6.069	1.042	-0.378	-5.823	0.000	-8.124 to -4.013
	Infant sex (ref: male)	-1.425	0.978	-0.095	-1.457	0.147	-3.355 to 0.504
	Model fit	R=0.441, R^2^=0.194; Adj. R^2^=0.182; F(4.195)=15.760; p<0.001					
Birth satisfaction scale	Constant	90.765	6.082		14.923	<0.001	
	Receiving information about pregnancy and birth process (ref: no)	7.965	1.645	0.323	4.843	<0.001	4.721–11.208
	Attendance at childbirth preparation classes (ref: no)	-6.378	2.752	-0.155	-2.318	0.022	-11.805 to -0.951
	Model fit	R=0.364, R^2^=0.133; Adj. R^2^=0.119; F(3.196)=9.995; p<0.001					

B: unstandardized regression coefficient; β: standardized regression coefficient; CI: confidence interval. SE: standard error.

*Negative β values indicate lower scores compared to the reference category.

A moderate and statistically significant correlation was found between women’s total PRMCS scores and total BSS scores (r=0.464, p<0.001). Furthermore, a significant relationship was determined between the total BSS score and all subscales of the PRMCS (providing comfort r=0.418, p<0.001; mistreatment r=0.441, p<0.001; participatory care r=0.369, p<0.001).

## DISCUSSION

Findings from the present study indicate that most women reported favorable perceptions of RMC throughout the childbirth experience. Similar findings have been reported in studies conducted in BFH across different regions of Turkey, which align with the results of the present study^8,9,13^. By contrast, research carried out in healthcare facilities without BFH designation has documented less favorable perceptions of RMC among women^14^. The positive findings of the present study may be partly attributed to the BFH status of the institution where the research was conducted. To create positive childbirth environments, it is essential to implement global-level decisions and prioritize RMC within national health policies. BFH aims to provide childbirth environments free from mistreatment, prioritize maternal comfort, and promote respectful and supportive care practices by healthcare professionals.

The study found that women’s family type, whether their pregnancies were planned, and their delivery methods were related to their perception of RMC. In patriarchal societies such as Turkey, women living in extended family structures may experience reduced autonomy and decision-making power during pregnancy and childbirth. Previous studies reported that planned pregnancies enable women to approach the birthing process more prepared and informed, and contribute to more positive perceptions of healthcare services^6,15^. Planned pregnancies are often associated with greater psychological readiness, increased engagement in antenatal care services, and more frequent communication with healthcare professionals, all of which may contribute to more positive perceptions of childbirth care. While the explanatory power of the regression models was limited, this outcome is in line with evidence suggesting that perceptions of maternity care are shaped by a wide range of interrelated factors. Interpersonal interactions, institutional culture, healthcare providers’ attitudes, and women’s pre-existing expectations may all play critical roles in shaping perceptions of RMC.

The present study found that women were generally satisfied with their childbirth experiences. However, studies conducted in different countries using the BSS have reported considerable variation in women’s levels of birth satisfaction^4,5,6,16,17^. These discrepancies may be attributed to the wide range of factors influencing birth satisfaction. In this study, education level and family type were identified as significant predictors of birth satisfaction. We considered that this finding might be related to lower expectations of care, the hospital-based data collection setting, and women’s reluctance to express dissatisfaction. Conversely, other studies have reported no significant association between educational level and birth satisfaction^18,19^, indicating that the relationship between education and birth satisfaction may vary across contexts. Furthermore, our findings indicated that women who received information about pregnancy and childbirth and participated in childbirth preparation classes reported higher levels of birth satisfaction. Similarly, Bal et al. found that women who received antenatal information and attended pregnancy education programs had significantly higher levels of birth satisfaction^17^. Although the explanatory power of the models predicting birth satisfaction was relatively limited, this finding aligns with previous research suggesting that birth satisfaction is influenced by a broad range of psychosocial, emotional, and contextual factors beyond sociodemographic and obstetric characteristics. Prior studies have emphasized that subjective childbirth experiences are not determined solely by clinical or structural variables but are also strongly shaped by women’s expectations, the emotional support they receive, and their perceived autonomy during childbirth^17,18,19,20^.

In the present study, women who reported higher perceptions of RMC also reported higher levels of birth satisfaction. Similarly, prior studies have consistently underscored the strong influence of factors such as information provision, respect for privacy, shared decision-making, and physical and ­psychological support on women’s satisfaction with childbirth^3,10,20,21,22^. Taken together, the findings of the present study and those of previous research indicate that childbirth is a multidimensional experience, and the relationship between perceptions of care and satisfaction depends on the overall quality of care provided. The moderate positive association observed between perceptions of RMC and birth satisfaction supports person-centered care theories, which emphasize dignity, autonomy, and supportive communication as key determinants of positive healthcare experiences. When women feel respected, adequately informed, and actively involved in decision-making during childbirth, they are more likely to perceive the birth experience as positive and satisfying, regardless of clinical outcomes.

This study was conducted in a single center and within a BFH setting; therefore, the generalizability of the findings to other institutions and regions is limited. Consequently, the levels of perceived RMC and birth satisfaction observed in this study may be higher than those in non-BFH settings, which may further restrict the generalizability of the results. Due to the cross-sectional design of the study, causal relationships between variables cannot be inferred. Additionally, data were collected within the first 24 h postpartum, and women’s physical and emotional conditions during this early period may have influenced their perceptions of care and satisfaction levels. The use of self-report measures also introduces the potential risk of social desirability and response bias.

## CONCLUSION

This study demonstrated that women’s perceptions of RMC were generally high and were significantly associated with birth satisfaction. The findings highlight the need for ongoing professional development initiatives that strengthen healthcare providers’ competencies in RMC and effective communication. Expanding access to educational resources related to pregnancy and childbirth and increasing the availability of childbirth preparation classes may help women engage more consciously and confidently in the childbirth process. Furthermore, maternity units should implement physical and organizational arrangements that protect privacy, encourage the presence of companions during labor and birth, and prioritize woman-centered care approaches. Finally, further studies involving larger samples and diverse regions are recommended to re-examine the relationship between RMC and birth satisfaction and to enable more comprehensive comparisons at the national level.

## Data Availability

The datasets generated and/or analyzed during the current study are available from the corresponding author upon reasonable request.
